# A rare cause of forearm pain: anterior branch of the medial antebrachial cutaneous nerve injury: a case report

**DOI:** 10.1186/1749-7221-3-10

**Published:** 2008-04-21

**Authors:** Necmettin Yildiz, Füsun Ardic

**Affiliations:** 1 Department of Physical Medicine and Rehabilitation, Faculty of Medicine, Pamukkale University, Denizli, Turkey

## Abstract

**Introduction:**

Medial antebrachial cutaneous nerve (MACN) neuropathy is reported to be caused by iatrogenic reasons. Although the cases describing the posterior branch of MACN neuropathy are abundant, only one case caused by lipoma has been found to describe the anterior branch of MACN neuropathy in the literature. As for the reason for the forearm pain, we report the only case describing isolated anterior branch of MACN neuropathy which has developed due to repeated minor trauma.

**Case presentation:**

We report a 37-year-old woman patient with pain in her medial forearm and elbow following the shaking of a rug. Pain and symptoms of dysestesia in the distribution of the right MACN were found. Electrophysiological examination confirmed the normality of the main nerve trunks of the right upper limb and demonstrated abnormalities of the right MACN when compared with the left side. Sensory action potential (SAP) amplitude on the right anterior branch of the MACN was detected to be lower in proportion to the left. In the light of these findings, NSAI drug and physical therapy was performed. Dysestesia and pain were relieved and no recurrence was observed after a follow-up of 14 months.

**Conclusion:**

MACN neuropathy should be taken into account for the differential diagnosis of the patients with complaints of pain and dysestesia in medial forearm and anteromedial aspect of the elbow.

## Introduction

The medial antebrachial cutaneous nerve (MACN) arises from the medial cord (78%) and the lower trunk (22%) of the brachial plexus. It goes along the course of the median and ulnar nerves, vena basilica, and arteria brachialis, in the upper arm [1]. In the literature, causes of MACN neuropathy include iatrogenic reasons such as steroid injection due to medial epicondylitis, routine venipuncture, cubital tunnel surgery, loose body removal, elbow arthroscopy, open fractures fixation, tumour excision, and arthrolysis [2-7]. It is also caused more rarely by repeated minor trauma (from tennis) and soft tissue laceration. It is even more rarely brought about by tuberculoid leprosy neuritis or subcutaneous lipoma [8-10]. However, MACN neuropathy is thought to be noticed less often due to the fact that it causes minor discomfort for the patients and does not affect the hand [10]. Although in some cases where MACN neuropathy was diagnosed, it was not specified which branch of the nerve was affected [3,7,9]. Due to the variety in its anatomic localization, the posterior branch of MACN is inclined to be more vulnerable to iatrogenic causes such as cubital tunnel surgery and direct invasive procedures to the medial part of the elbow [2,4-6,11]. Although the cases in the literature describing neuropathy of the posterior branch of the MACN are abundant [2,4-6] only one case caused by lipoma has been found to describe the anterior branch of the MACN as the site of neuropathy [10]. As for the reason for forearm pain, we report the only case describing isolated neuropathy of the anterior branch of the MACN which has developed due to repeated minor trauma.

## Case presentation

A 37-year-old woman patient who is a homemaker was accepted to our hospital with the complaint of a 10-day pain in her right upper limb. She mentioned that the pain first involved the elbow and then the forearm, particularly the medial part of it. Nearly 10 days before, while she was cleaning and shaking the rug, she developed a discomforting pain in her right elbow. She explained that the pain in her elbow had become worse and in 24 hours spread through her whole forearm. She added that, previously, the pain had been partially responding to NSAI drugs, but subsequently, it continued to progressively increase.

**Figure 1:**
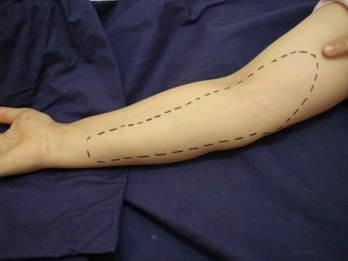
The view of dysesthesic region.

There was a pain in her medial forearm and elbow. She felt abnormal when she was palpated on her medial forearm. During her examination, she was able to describe the point where her pain started in the proximal part of her elbow. On detailed neurological examination, a region of dysesthesia which extends from the elbow to the medial forearm was detected (Figure 1). The patient had no history of polyneuropathy, chronic systemic disease, injection or surgical intervention at the elbow. Range of motion, motor, and reflex examinations of both upper extremities were normal. Cervical spine examination was normal. Varus-valgus stress test for the elbow was normal. Medial epicondylitis test and tinel test for the ulnar nerve were negative.

X-ray views of the elbow, including oblique views, appeared normal. Electromyography showed normal findings in the right biceps, triceps, flexor digitorum sublimis, pronator quadratus, interosseous and abductor pollicis brevis muscles, and nerve conduction studies in both upper limbs except for the right MACN were found normal. The MACN is stimulated antidromically at the lateral border of the biceps brachii tendon in the cubital fossa. An active surface recording electrode is placed on the anteromedial surface of the forearm 14 cm from the active stimulating electrode. Sensory action potential (SAP) amplitude of the right anterior branch of the MACN was detected to be lower in proportion to the left. The sensory conduction velocity (SCV) was normal. On both right and left sides, the posterior branch of the MACN was normal and symmetrical for amplitude and velocity (Table 1). On magnetic resonance imaging of the elbow, no lesion was detected which may cause MACN neuropathy.

**Table 1:** The nerve conduction data of the case.

	RIGHT		LEFT	
**MACN**	**SCV (m/s)**	**AMP (μV)**	**SCV (m/s)**	**AMP (μV)**

**Anterior Branch**	57	2	56	9
**Posterior Branch**	56	10	58	11

**MACN: **Medial Antebrachial Cutaneous Nerve.

**SCV: **Sensory Conduction Velocity.

**AMP: **Sensory Action Potential (SAP) Amplitude.

As well as NSAI drug treatment, physical therapy of 15 days (TENS, ultrasound, ROM exercises) was applied to the patient. The complaint of pain was totally relieved. Two months later, the dysesthesia disappeared. No recurrence occured after a follow-up of 14 months.

## Conclusion

Although isolated MACN neuropathy may be caused by various iatrogenic reasons, it is described rarely by the reasons of repeated minor trauma or soft tissue laceration [6,8]. In the study by Stahl and Rosenberg, 12 patients with MACN neuropathy were described. In two patients, the reason for neuropathy was stated to be soft tissue laceration but the shape and the cause of the injury was not described [6]. Chang and Ho reported that MACN neuropathy described in one of their cases was not isolated, but was assosiated with lesion of the median nerve, and that the reason for a second case with isolated MACN neuropathy was repeated minor trauma [8]. In the literature, the reason for the only case stating that the anterior branch of the MACN was damaged was lipoma [10]. Our case, however, is the only case describing isolated neuropathy of the anterior branch of the MACN which was developed by repeated minor trauma. Shaking a rug is a specific method of cleaning the rug in which the elbows and wrist will be used in repetitive flexion and extension. This activity requires forceful sustained contraction of the shoulder girdle, upper arm, and forearm muscles to hold the rug against the force of the weight of the rug and gravity. Because of the superficial location of the nerve adjacent to the biceps tendon, full extension of the elbow and repetetive forceful contracture of the flexor musculature may place this nerve under stretch, effectively bowstringing it across the elbow.

Both because it does not cause any limitation in the elbow and it can not be detected by the radiologic MR imaging, the neuroma is marginalized. Seror stated that the lesions of MACN are rarely seen because we do not notice them for several reasons such as isolated lesions of MACN not affecting the hands, their causing only minor discomfort, and the electrophysiological studies of MACNs not being part of routine upper extremity electrodiagnostic examinations [10]. Izzo et al. noted that in addition to the median nerve sensory studies, the forearm sensory nerve examinations can also be used to detect the situations of peripheral neuropathy, brachial plexopathy and local neuropathy [12]. MACN conduction studies were performed by Seror in 70 control subjects to determine normal values and define the lower limits of normality. The mean SAP amplitude was 17.5 μV, and the SCV was 61 m/s. In the same study no SAP amplitude was lower than 6 μV [13]. With reference to the reported normal conduction values and the studies by Chang and Ho, and by Seror, our case was diagnosed with right MACN neuropathy due to the detections of normal SCV and lower SAP amplitude of the right MACN [8,10,12,13] (Table 1).

Any surgical intervention, injection, trauma or forcing activity of the elbow should be questioned and nerve neuropathies should be considered, though they are rare, for the complaints of forearm pain.

In conclusion, especially for the patients with complaints of pain and dysesthesia in the medial forearm and anteromedial aspect of the elbow, MACN neuropathy should be taken into account for the differential diagnosis and, therefore, electrophysiologic examination should be performed.

## Competing interests

The authors declare that they have no competing interests.

## Authors' contributions

NY and FA contributed equally to this case report. All authors read and approved the final manuscript

## Consent

Written informed consent was obtained from the patient for publication of this case report and any accompanying images.
